# Protective Role of *Mytilus edulis* Hydrolysate in Lipopolysaccharide-Galactosamine Acute Liver Injury

**DOI:** 10.3389/fphar.2021.667572

**Published:** 2021-05-18

**Authors:** Eleonora Starikova, Jennet Mammedova, Arina Ozhiganova, Aleksandra Lebedeva, Anna Malashicheva, Daria Semenova, Evgeniia Khokhlova, Eleonora Mameli, Andrea Caporali, Jimi Wills, Alexey Sokolov

**Affiliations:** ^1^ Laboratory of Immunoregulation, Department of Immunology, Institute of Experimental Medicine, St. Petersburg, Russian Federation; ^2^ Laboratory of General Immunology, Department of Immunology, Institute of Experimental Medicine, St. Petersburg, Russian Federation; ^3^ Laboratory of Molecular Cardiology, Almazov National Medical Research Centre, St. Petersburg, Russian Federation; ^4^ Laboratory of Regenerative Biomedicine, Institute of Cytology RAS, St. Petersburg, Russian Federation; ^5^ Laboratory of Vascular Biology, University/BHF Centre for Cardiovascular Science, University of Edinburgh, Edinburgh, United Kingdom; ^6^ Cancer Research United Kingdom Edinburgh Centre, Institute of Genetics and Cancer, University of Edinburgh, Edinburgh, United Kingdom; ^7^ Laboratory of Biochemical Genetics, Department of Molecular Genetics, Institute of Experimental Medicine, St. Petersburg, Russian Federation

**Keywords:** *M. edulis* hydrolysate, acute liver injury, vascular permeability, nitric oxide, VCAM-1, IL-6

## Abstract

Acute liver injury in its terminal phase trigger systemic inflammatory response syndrome with multiple organ failure. An uncontrolled inflammatory reaction is difficult to treat and contributes to high mortality. Therefore, to solve this problem a search for new therapeutic approaches remains urgent. This study aimed to explore the protective effects of *M. edulis* hydrolysate (N2-01) against Lipopolysaccharide-D-Galactosamine (LPS/D-GalN)-induced murine acute liver injure and the underlying mechanisms. N2-01 analysis, using Liquid Chromatography Mass Spectrometry (LCMS) metabolomic and proteomic platforms, confirmed composition, molecular-weight distribution, and high reproducibility between *M. edulis* hydrolysate manufactured batches. N2-01 efficiently protected mice against LPS/D-GalN-induced acute liver injury. The most prominent result (100% survival rate) was obtained by the constant subcutaneous administration of small doses of the drug. N2-01 decreased Vascular Cell Adhesion Molecule-1 (VCAM-1) expression from 4.648 ± 0.445 to 1.503 ± 0.091 Mean Fluorescence Intensity (MFI) and Interleukin-6 (IL-6) production in activated Human Umbilical Vein Endothelial Cells (HUVECs) from 7.473 ± 0.666 to 2.980 ± 0.130 ng/ml *in vitro*. The drug increased Nitric Oxide (NO) production by HUVECs from 27.203 ± 2.890 to 69.200 ± 4.716 MFI but significantly decreased inducible Nitric Oxide Synthase (iNOS) expression from 24.030 ± 2.776 to 15.300 ± 1.290 MFI and NO production by murine peritoneal lavage cells from 6.777 ± 0.373 µm to 2.175 ± 0.279 µm. The capability of the preparation to enhance the endothelium barrier function and to reduce vascular permeability was confirmed in Electrical Cell-substrate Impedance Sensor (ECIS) test *in vitro* and Miles assay *in vivo*. These results suggest N2-01 as a promising agent for treating a wide range of conditions associated with uncontrolled inflammation and endothelial dysfunction.

## Introduction

Septic shock is a massive uncontrolled inflammatory reaction accompanied by excessive production of inflammatory cytokines and violation of vascular homeostasis, manifested as hypotension and peripheral vasodilation. The subsequent collapse of blood circulation, refractory to intravascular volume replacement and vasopressors, leads to hypoperfusion of organs followed by multiple organ failure (Shapiro et al., 2006). Neither the significant progress made in the study of the sepsis pathophysiology nor the use of various strategies for the treatment of septic shock has allowed any significant improvement in survival in this pathology (Berg and Gerlach 2018). The frequency of immune system disorders with pro-and anti-inflammatory cytokine production impairment and systemic inflammatory response syndrome (SIRS) development remains significantly high, accounting for 40% of deaths in intensive care units (Punyadeera et al., 2010; Vincent et al., 2019). Therefore, the development of new therapeutic drugs that can suppress an excessive inflammatory reaction without compromising the immune system’s main protective mechanisms remains relevant. Marine fauna is a resource providing great opportunities to develop new biopharmaceuticals (Ahmad et al., 2019). *Mytilus edulis* (*M. edulis*) is a typical marine bivalve that inhabits coastal rocks. *M. edulis* derivatives were shown to contain biologically active substances that can effectively regulate inflammation (Kim et al., 2016; Cheonge et al., 2017; Lindqvist et al., 2018), blood clotting (Jung and Kim, 2009; Feng et al., 2018; Qiao et al., 2018) and oxidative stress (Wang et al., 2013; Grienke et al., 2014). *M. edulis* broth sauce is traditionally used in China to enhance immune reactions and treat liver and kidney dysfunctions (Li and Ding, 2006). But the usage of *M. edulis* derivatives against the SIRS has not yet been investigated and needs to be studied. Therefore, the present study aims to look into the possible protective role of *M. edulis* hydrolysate (N2-01) in murine model Lipopolysaccharide-D-Galactosamine (LPS/D-GalN) acute liver injury *in vivo*, and the underlying mechanisms *in vitro*.

## Materials and Methods

### Preparation and Characterization of *M. edulis* Hydrolysate

*M. edulis* for N2-01 preparation were harvested on the west coast of Scotland and the Shetland Islands; alive mussels were adequately processed, the mussels’ meat composition of each lot was analyzed, and stored at −70°C. N2-01 was produced from the standardized composition of mussel meat through a patented (EP2911678B1) hydrolysis process. The sustainability of the lab-scale pre-GMP production was confirmed by the IGMM (Institute of Genetics and Molecular Medicine) Mass Spectrometry laboratory (University of Edinburgh). N2-01 (in its undiluted form) was diluted 40:1 in 5:3:2 methanol:acetonitrile:water and 10 ul were injected onto a ZIC-pHILIC 4.6 mm x 150 mm Sequant column (Merck, United Kingdom) on an Ultimate 3000 series HPLC (Thermo Fisher Scientific, United States) with A gradient from 90 to 5% B in 20 min, where A was 20 mm ammonium carbonate and B was acetonitrile. Data were acquired on Q Exactive (Thermo Fisher Scientific, United States) with HESI in positive (75.5–1132.5) and negative (77.5–1132.5) mode at 70k resolution, with ddMS2 at 17.5k. Quantitative comparisons and primary identification were done in Compound Discoverer 2.1 (Thermo Scientific). Further identification was done in PEAKS 7.5 (Bioinformatics Solutions Inc.)

### Animals

8 week-old male C57BL/6 mice, white mongrel female and CBA/BALB male (F1) mice (all received from “Rappolovo” nursery, St. Petersburg, RF) were housed at 24 ± 1°C, a 12 h light-dark cycle and relative humidity of about 40–80% conditions. Animal experiments were carried out at the Institute of Experimental Medicine, St-Petersburg, according to Animal Welfare Assurance №2/19 from March 25, 2019.

### Murine Model of Lipopolysaccharide/D-Galactosamine Acute Liver Injury

C57BL/6 mice were used in the experiments. The study was carried out as described in (Galanos et al., 1979).

Each mouse was intraperitoneally administered 500 μl (25 ml/kg) mixture, containing 200 ng (0.01 mg/kg) of LPS from *E. coli*, strain O26: B6 (No. L-2762, Sigma-Aldrich, United States) and 5 mg (250 mg/kg) of D-GalN (Vekton, RF). Saline was used for sham treatment. The experiment was carried out using four groups of animals. The test group received 0.1 (5 ml/kg or 20 ml/kg per day), 0.2 (10 ml/kg or 40 ml/kg per day) and 0.4 (20 ml/kg or 80 ml/kg per day) ml N2-01 four times per day intraperitoneally at 8:00 AM, 12:00 PM, 4:00 PM and 8:00 PM (36, 36 and 12 mice were included in each group respectively). The control group (36 mice included) received sterile saline at the same time points. A single dose of 5 mg (250 mg/kg) of antibodies against the macrophages migration inhibiting factor (MIF) in 0.4 ml (20 ml/kg) of the physiological solution was used as the active control (6 mice included).

An additional experiment with eight animals in each group was performed with the subcutaneous implantation of Alzet osmotic pumps (Models 1003D, Nominal Pumping Rate 1.0 μl/h (0.05 ml/kg per hour or 1.2 ml/kg per day). Nominal Duration 3 days. Nominal Reservoir 100 μl, Lack (DURECT Corporation, Cupertino, CA) containing 0.1 ml (5 ml/kg) of N2-01 (test group) or physiological solution (control group). Osmotic Pumps implantation was performed as described in (Krogman et al., 2016). The mortality rate was recorded every 24 h and Kaplan-Meier’s plots were created.

### Miles Assay: Vascular Permeability

The level of vascular permeability was assessed in white mongrel female mice using Evans blue dye as previously described (Brash et al., 2018). 22 animals in each group studied. To determine extravascular protein leakage *in vivo*, Evans blue dye (0.2 ml (10 ml/kg) of 0.2% solution in PBS) (DIA-M, RF), which can bind quantitatively to serum albumin, was injected intravenously through the tail vein. After 10 min, mice were anaesthetized using isoflurane (AbbVie Inc., United Kingdom) inhalation. Prior to vascular hyperpermeability stimulation, 100 µl (5 ml/kg) of N2-01 was injected in the withers subcutaneously. Animals of the control group were administered with saline instead of N2-01 in the same manner. Then 20 µl (1 ml/kg) of compound 48–80 known to induce vascular leakage (Ashina et al., 2015), in concentration 3 μg/ml was administered intradermally (Sigma-Aldrich, United States) in the right flank. Simultaneously, N2-01 (test group) or PBS (control group) were injected subcutaneously into the withers. The animals were euthanized by cervical dislocation in 20 min after the Evans blue injection. The skin regions comprising the leakage of Evans blue dye at the site of permeability-inducing agent injection were excised using punch. The skin samples were stored at −20°C until further use. Evans blue was extracted from the skin by incubation in 0.2 ml of formamide (Sigma-Aldrich, United States) for 24 h, centrifuged 5.000 g for 10 min 0.1 ml of the supernatant of each sample were collected into flat-bottom 96-well plates (Sarstedt, Germany). The concentration of Evans blue was quantified by measuring the absorbance at 620 nm using a Microplate Reader (BioRad, United States). The results were expressed as optical density.

### Isolation of Human Umbilical Vein Endothelial Cells

Endothelial cells from human umbilical veins were isolated as previously described (Baudin et al., 2007). Umbilical cords were obtained from the perinatal center of the Almazov National Medical Research Center. Ethics committee of the Almazov National Medical Research center approved the research protocol for the study “Investigation of cellular and molecular bases of aortic pathologies using tissue obtained from the leftovers after surgical interventions”. Ethical permit number December 26, 2014. The form of uniformed content for the patients enrolled in the study is approved by Ethics committee of the Almazov National Medical Research center. Primary cultures of human umbilical vein endothelial cells (HUVEC) were grown in Endothelial Cell Basal Medium-2 (ECBM-2) (Promocell, Germany) with Supplement Mix (Promocell, Germany), 10% fetal calf serum (FCS) (HyClone, United States), 4 mM glutamine, 50 µg/ml penicillin, 50 µg/ml streptomycin in tissue culture flasks (Sarstedt, Germany) which had been pre-coated with 0.2% gelatin (Sigma, United States) at 37°C in a humidified atmosphere with 5% CO_2_. Subculturing was performed twice a week. The monolayer disintegration was caused by cells incubation in Trypsin-EDTA solution (Sigma, United States). Cells from three to five passage were used in experiments.

### The Assessment of Nitric Oxide Production by Human Umbilical Vein Endothelial Cells

To analyze NO production, HUVECs were plated into 12-well flat-bottom plates (Sarstedt, Germany) and cultured to form a confluent monolayer. After that, N2-01 was added, and cells were incubated 37°C in a humidified atmosphere of 5% CO_2_. LPS from *E. coli* O111:B4 (Sigma-Aldrich, United States) in concentration 100 μg/ml was added for the last 24 h of incubation. At the end of the incubation period, the DAF-FM DA dye (Invitrogen, United States) was added at a concentration of 1 µM in each well. After 1 h incubation, the dye was washed off, the monolayers were disintegrated, and the cells were fixed with a 4% formaldehyde solution (Sigma, United States of America). Here and further, the samples for flow cytometry were analyzed using a Navios ™ flow cytometer (Beckman Coulter, United States). The results were expressed as Mean Fluorescence Intensity (MFI).

### Real-Time Quantitative Polymerase Chain Reaction (PCR)

RNA from cultured cells was isolated using ExtractRNA (Eurogene, RF). Total RNA (0.5 μg) was reverse transcribed with MMLV RT kit (Eurogen, RF). Real-time Quantitative PCR was performed with 1 μl cDNA and SYBRGreen PCRMastermix (Eurogen, RF) in the Light Cycler system using specific forward and reverse primers for target genes. Corresponding gene expression level was normalized to GAPDH from the same samples. Changes in target genes expression levels were calculated as fold differences using the comparative ΔΔCT method. Primer sequences were.

human ICAM-1:
F - 5’ - CGG​CCA​GCT​TAT​ACA​CAA​GAA​C- 3′, R - 5’ - TGG​CAC​ATT​GGA​GTC​TGC​TG - 3’;
human VCAM-1:
F 5’ -CAG​TAA​GGC​AGG​CTG​TAA​AAG​A - 3′, R 5’ - TGG​AGC​TGG​TAG​ACC​CTC​G - 3’;
human IL-6:
F 5’ - GCT​CTG​TGT​GAA​GGT​GCA​GTT - 3’
R 5’ - GTG​GTC​CAC​TCT​CAA​TCA​CTC​T - 3’.

### Analysis of Endothelial Cells Adhesion Molecules Expressions

To evaluate the expression of VCAM-1 (CD106) and ICAM-1 (CD54) adhesion molecules, HUVECs were seeded into 24-well flat-bottom plates (Sarstedt, Germany) at a concentration of 150,000 cells per ml. Then, the N2-01 in different dilutions was added for 72 h. 24 h before the end of incubation, 100 μg/ml LPS from *E. coli* O111:B4 (Sigma-Aldrich, United States) was added into the test wells, and the same volume of vehicle was added into the control wells. The expression of surface molecules was evaluated by flow cytometry using phycoerythrin (PE) labelled anti-CD106 (Beckman Coulter, United States, cat. No. PN A66085), anti-CD54 monoclonal antibodies (Beckman Coulter, United States, cat. № PN IM1239U) and isotype control antibody mouse IgG1-PE (Becman Coulter, United States, Cat. No PN IM0670). Single-cell suspensions staining was performed following the manufacturer's recommendation. To exclude dead cells from the analysis, the cells were stained with 1 mg/ml DNA-binding dye DAPI (Invitrogen, United States).

### Analysis of Interleukin-6 Production by Human Umbilical Vein Endothelial Cells

HUVECs were seeded into 24-well flat-bottom plates (Sarstedt, Germany) at a concentration of 150,000 cells per ml and N2-01 in different dilutions with or without 50 U/ml TNFα (“Refnolin”,“Ferment”, Sanitas, Lithuania, specific activity - 1 U-0.06 ng) was added. After 24 h, culture medium samples were collected and stored at −20°C. The assessment of IL-6 concentration in the samples was performed using a human IL-6 ELISA kits (Cytokine, RF), following the manufacturer’s instructions.

### Analysis of Nitrite and Nitrate Concentrations in Mouse Peritoneal Lavage Cell Supernatants

The experiments were carried out with CBA/BALB (F1) mice. Experiments were performed as described previously (Migliorini et al., 1991). The cells were received by the wash of the peritoneal cavity with 5 ml Hanks solution supplemented with 2% FCS (HyClone, United States). After a single washing by centrifugation 5 min 200 g, cells were seeded into 96-well flat-bottom plates (Eppendorf, Germany) at the density of 300, 000 per well in 100 µl of RPMI 1640 medium (Biolot, RF) supplemented with 10% FCS (HyClone, United States), 2 mM glutamine (Biolot, RF), 50 μg/ml gentamicin (Biolot, RF) and incubated for 24 h in a humidified atmosphere 37°C, 5% CO_2_. After the culture medium replacement, the N2-01 was added in each well in different concentrations. To stimulate NO production, LPS from *E. coli* 055:B5 (Sigma-Aldrich, Germany) was added at 1 μg/ml. After 24 h of incubation at 37°C and 5% CO_2_, the cells were centrifuged for 5 min at 200 g. Next, 70 µl of the supernatants were transferred into 96-well flat-bottom plates (Eppendorf, Germany), and 70 µl of Griess reagent in each well was added. The spectrometric analysis was performed at a wavelength of 540 nm (Microplate reader, Model 680, Bio-Rad). The concentration of nitrites and nitrates in experimental samples was determined statistically, in accordance with a linear approximation using the least-squares method, based on a calibration curve constructed using a solution of sodium nitrite (NaNO_2_) of the known concentration. Cellular precipitations obtained were used for subsequent determination of the level of iNOS expression. Experiments were performed in triplicates with six animals in each repeat.

### Analysis of Inducible Nitric Oxide Synthase Expression in Peritoneal Lavage Cells

Peritoneal lavage cells were transferred into tubes for flow cytometry (Sarstedt, Germany). Fixation/permeabilization was performed by incubation in 500 µl in ice-cold 80% methanol (Vecton, RF) for 10 min at −20°C. After a single wash by centrifugation at 200 g for 7 min, the cell suspension was stained with APC/Cy7 labelled anti-CD45 monoclonal antibodies (Biolegend, United States cat. No.103116) or isotype control antibody rat IgG2b APC/Cy7 (Biolegend, United States, Cat. No 147718), and FITC labelled monoclonal antibodies against iNOS (BD Transduction Laboratories, United States Cat. No. 610330), following manufacturer's recommendations. CD45—positive cells were included in the analysis.

### Analysis of Endothelial Monolayer Barrier Function Using Electrical Cell-Substrate Impedance Sensor

Endothelial barrier function was continuously recorded using the 8W10E + electrode chamber arrays and ECIS Z-Theta system (both Applied Biophysics, United States) with associated software, as described in (Tiruppathi et al., 1992). Human brain endothelial cells (purchased from CellBiologics, United States) were cultured in Complete Human Endothelial Cell Medium (CellBiologics, United States), plated in fibronectin-coated (Merck, United Kingdom; 10 μg/ml) 8W10E + array, and grown to confluency to form an endothelial monolayer. The cells were pre-treated with N2-01 for 48 h and then stimulated with LPS from *E. coli* O111:B4 (Merks, United Kingdom; 1 mg/ml) for 24 h. The capacity of N2-01 to restore barrier function in response to LPS has been monitored and recorded for 24 h.

### Statistical Analysis

Kolmogorov-Smirnov test was used to confirm the normality of the distribution. In the studies of the production of NO and IL-6, the expression of iNOS and adhesion molecules, the ICAM-1, VCAM-1 and IL-6 transcripts expression, as well as the barrier function of the endothelial monolayer, the differences between test and control groups were estimated using one-way analysis of variance (ANOVA). Group-wise comparisons were performed with post hoc Tukey HSD test. Survival of mice in of D-GalN/LPS-induced liver injury was compared using the Log-rank test for trend. Differences in vascular permeability in the control and experimental groups of animals in Miles assay were estimated using Student’s t-test. Statistical analysis was performed using STATISTICA 7.0, Graph Pad Prism and Microsoft Office Excel 2010 software and a value of *p* < 0.05 was considered statistically significant.

## Results

### Composition and Reproducibility of Hydrolysate

**FIGURE 1:**
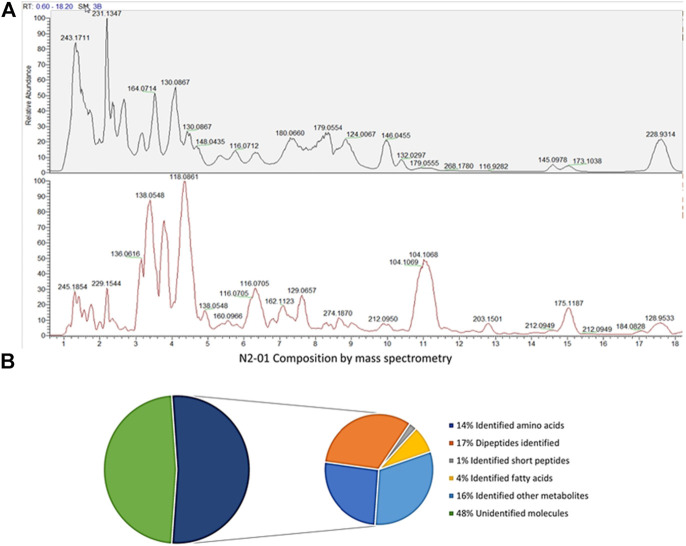
N2-01composition by mass-spectrometry. **(A)** Percentage of total ion intensity explained by Compound Discoverer and PEAKS for production batches 4, 5 and 6 combined. **(B)** Chromatogram of batch 6, negative mode top, positive mode bottom.

LCMS `omic analysis of N2-01 revealed an overview of its molecular composition and molecular-weight distribution and confirmed high reproducibility between manufactured batches. HILIC LC-MS/MS, performed in positive and negative modes, revealed a complex mixture of components, mostly peptides. Protein assay and standard proteomics indicated no measurable proteins remain after hydrolysis (data not shown). Compound discoverer identified 9473 unique molecular weights from the ion maps, suggesting molecular formulae for 4253 of these and giving unequivocal matches to 498 named compounds. PEAKS matched 1822 peptides from the Uniprot bivalve sequences, though many more had spectra characteristic of peptides. Compound Discoverer gave a name to 46% of the total ion intensity in three analyzed batches, with greater than 13% of the total intensity being attributed to amino acids but less than 1.5% to dipeptides (Figure 1). Greater than 5% of the total intensity was attributed to fatty acids and more than 20% attributed to other named metabolites. Amino acids, dipeptides and short peptides were also measured by PEAKS. Short peptides were in the mass range 344–698 with a median of 389. PEAKS identified slightly more of the amino acid and peptide associated intensity than Compound Discoverer, and the values from PEAKS are used in Figure 1B. That is PEAKS assigned 14, 17 and 1.2% of the total ion intensity to amino acids, dipeptides and short peptide, respectively, indicating the extent of hydrolysis. 48% of the total ion intensity remains unassigned (Supplementary Tables S1, S2). Mass spectrometry confirmed the composition one might expect from such a hydrolysate.

**FIGURE 2:**
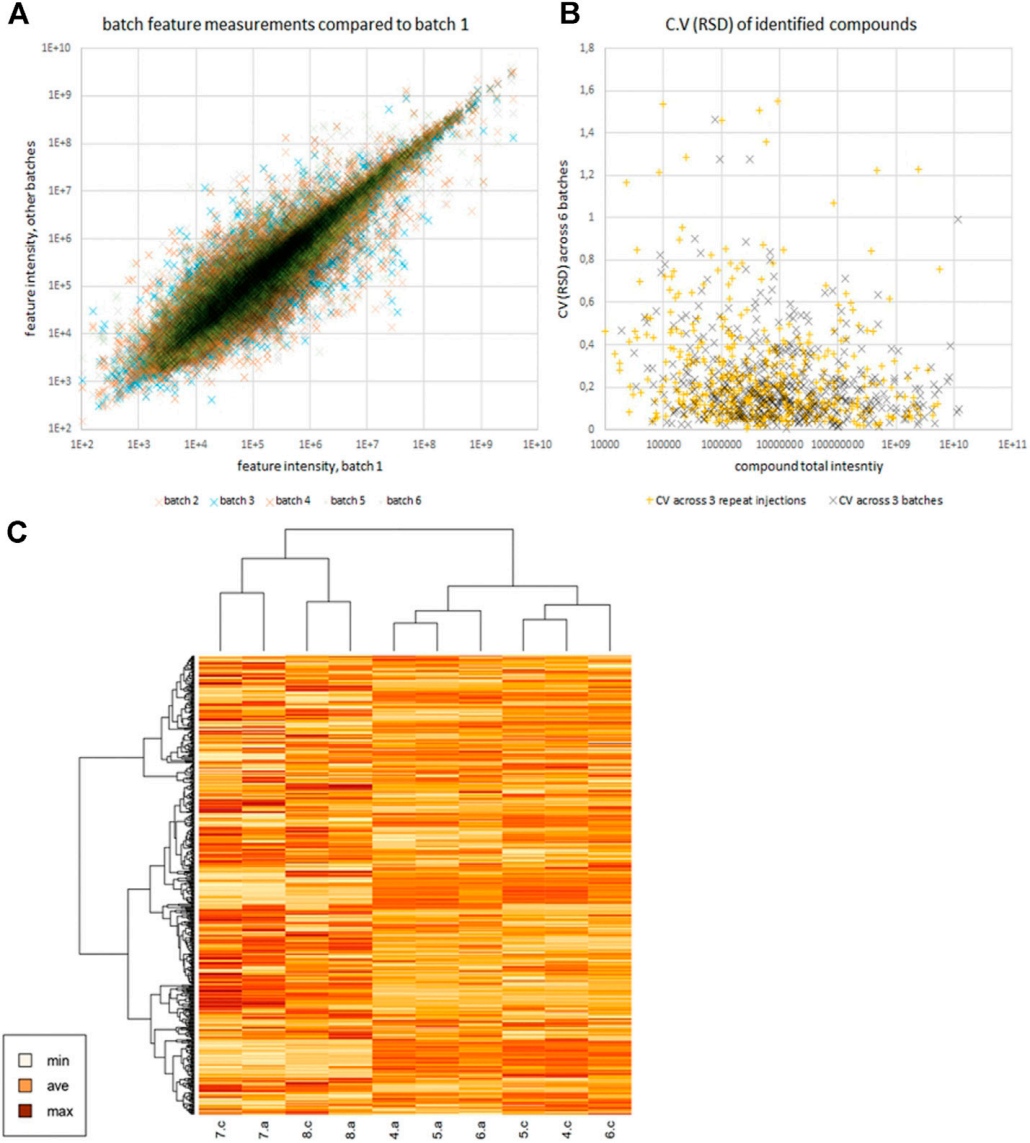
Panel **(A)** shows scatter plot of feature intensities of batches 2–6 vs batch 1, illustrating the reproducibility between batches. Panel **(B)** compares the CVs (RSDs) of identified compounds across repeat injections and across batches. Panel **(C)** is a heatmap generated in R version 4.0.0 (April 24, 2020) of scaled log intensity reported by Compound Discoverer for those compounds identified (mostly small molecules, not peptides). Compounds with multiple features were summed (Supplementary Table S3). Technical repeats, a and c are shown for N2-01 batches 4, 5 and 6, alongside 7 and 8 which are octopus and squid respectively.

Preparation of N2-01 was reproducible between batches. Focusing on the 5000 features most stable between repeat injections, from the most reproducible part of the HPLC gradient, repeat injections still accounted for 47.2% of the variability observed in the principal component analysis. Simultaneously, three batches (batches 4, 5, and 6) compared separated in component 2 with just 19.7%. The explained variance in PLS-DA was 22.9% in separating the batches, with component 2 having a variance 39.2%. Therefore, it is clear that batch production is very reproducible, to the extent that the vast majority of variation comes from sample-processing and measurement and not batch-to-batch variation, even when the selection of data favours repeat-injection stability. The reproducibility of batch preparation is illustrated in Figure 2A. The data are normally distributed when log-transformed, with a mean correlation coefficient of 0.92. Figure 2B shows that the CVs (RSDs) between batches are not very different from repeat injections, and the median CV (17%) is the same for both dimensions (Supplementary Table S4). Figure 2C shows comparison of batches, technical repeats, and contrasts *M. edulis* hydrolysates with those of other species. Note that while the different starting materials cluster apart, it is the technical repeats that cluster rather than batches of N2-01, further indicating that batch differences are minimal. LCMS data used in this paper are available at ftp://massive.ucsd.edu/MSV000087104/.

### Hydrolysate Increase Animals Survival Rate in the Murine Model Lipopolysaccharide/D-Galactosamine Acute Liver Injury

**FIGURE 3:**
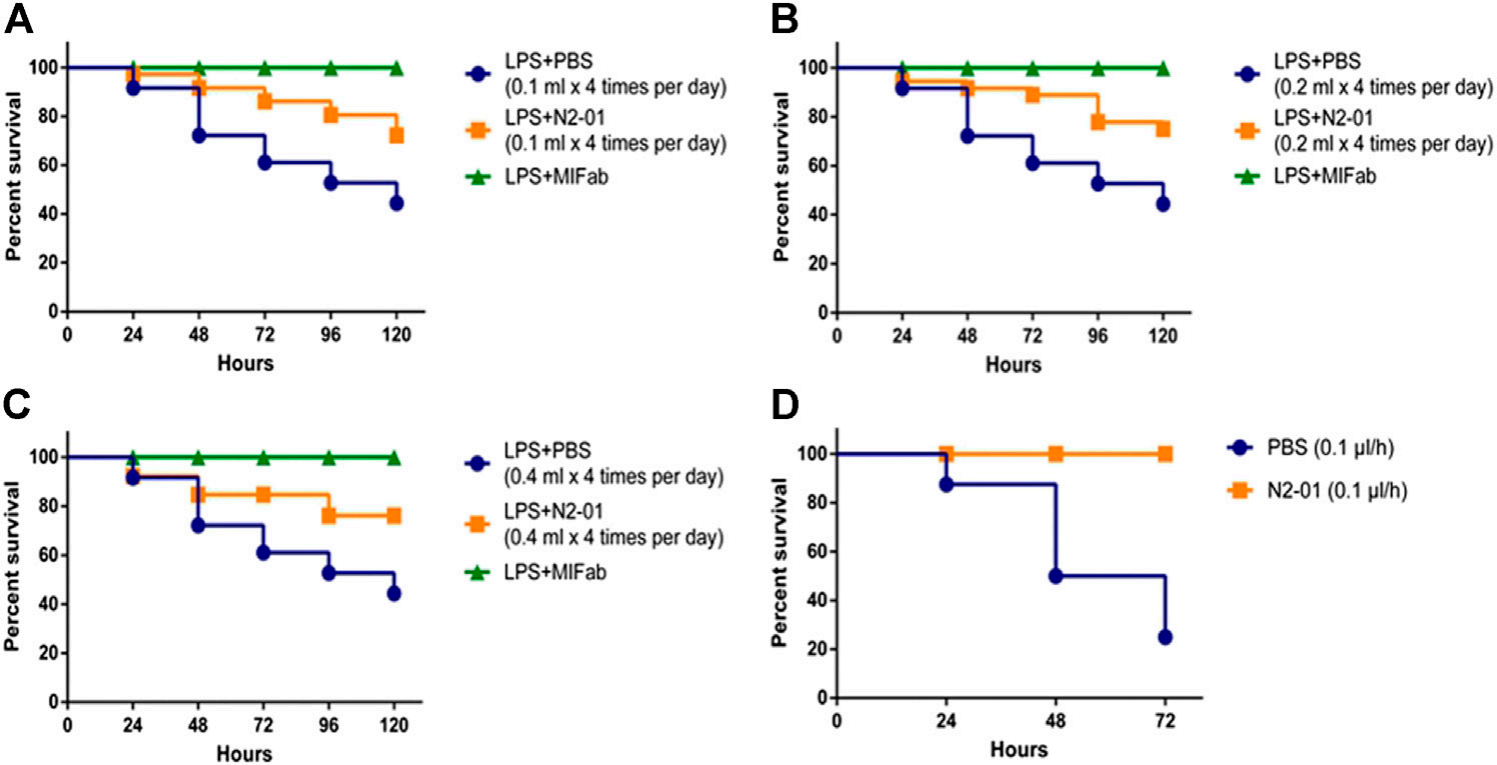
Effects of N2-01 treatment on LPS/GalN-induced lethality. **(A)** 0.1 ml N2-01 (4 time per day), **(B)** 0.2 ml N2-01 (4 time per day), **(C)** 0.4 ml N2-01 (4 time per day), **(D)** 1.0 μL/h N2-01 was infused with Alzet osmotic pumps. Group survival was compared using a Logrank test Logrank test for trend.

It was shown that 0.1 ml (5 ml/kg) N2-01 administered four times per day doubled the survival rate of the animals (Figure 3A). In comparison, the mortality in the control group was 50%, the mortality in the N2-01 group animals made up only 25%. It should be noted that the mortality rate in the test N2-01 group was registered in the morning (8:00 AM) before the administration of the next drug dose, rather than in the evening of the same day (the drug injection period). Therefore, it was suggested that either the N2-01 dose or the evenness of its administration during the day should be increased to enhance the effect. So, higher doses of the N2-01—0.2 (10 ml/kg) and 0.4 ml (20 ml/kg) were used in the next experiment. The increase in the drug dosage did not improve survival, and the mortality rate in the test groups (0.2 and 0.4 ml of N2-01) was nearly the same at all the time intervals (Figure 3A–C). At the next stage, the animals have implanted Alzet osmotic pumps that uniformly released 1.0 μl/h (0.05 ml/kg per hour or 1.2 ml/kg per day) of the drug in 3 days’ time after the implantation. 100% of the mice with Alzet pumps with the N2-01 implanted survived until the fourth observation day (Figure 3D), while in the control group, six of the eight mice died by the fourth day (i. e. the mortality rate was 75%).

### Hydrolysate Anti-Inflammatory Action *In Vitro*

**FIGURE 4:**
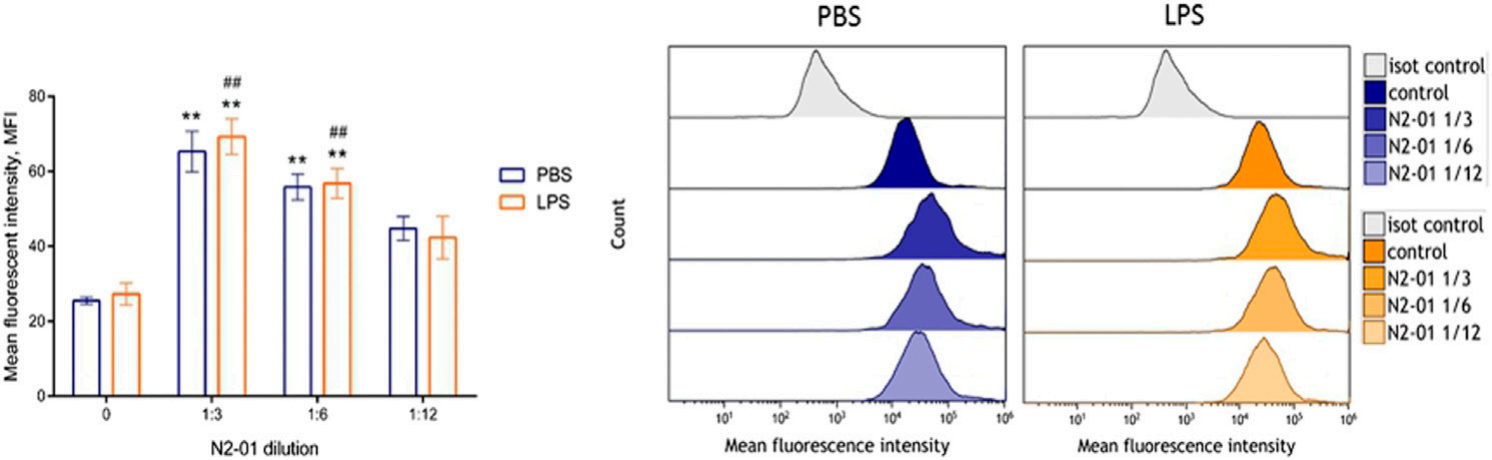
The effect of N2-01 on NO production by the human umbilical endothelial cells. Note. Statistical assessment of differences was performed using ANOVA (*p* < 0.001), and pairwise comparison of the mean values was performed using the Tukey HSD test. Data are expressed as mean ± standard error of the mean (SEM) of at least four individual experiments (*n* = 4). The differences are significant: **p < 0.01 vs PBS; ##*p* < 0.01 vs LPS.

It was shown that liver damage and systemic inflammatory reaction with the development of endothelial dysfunction play an important role in LPS/GalN-induced liver injury pathogenesis (Zhang et al., 2014). Liver damage is associated, in particular, with increased production of nitric oxide (NO) (Tsai et al., 2018), the product of NOS activity. At least three isoforms of this enzyme, which differ in function, cell expression, and regulation mechanisms were described (Sass et al., 2001). Of these, eNOS = NOS3 (endothelial NOS) is constitutively expressed by vascular endothelial cells and produces NO at low concentration to maintain vascular homeostasis (Sass et al., 2001). iNOS = NOS2—isoform is induced in immune cells under the influence of pro-inflammatory factors such as TNF-α, IFN-γ, LPS, etc. (Sass et al., 2001). iNOS overexpression has been described in many pathologies associated with the development of acute and chronic inflammation, including septic shock and hepatitis (Sass et al., 2001). To evaluate the action of the N2-01 on the activity of eNOS and iNOS isoforms of the enzyme, in further experiments, the effect of the drug on spontaneous and LPS-induced NO production by HUVECs and mouse peritoneal lavage cells were studied. It was shown that LPS did not affect the production of NO by endothelial cells (Figure 4). At the same time, N2-01 in all dilutions significantly increased the production of NO.

**FIGURE 5:**
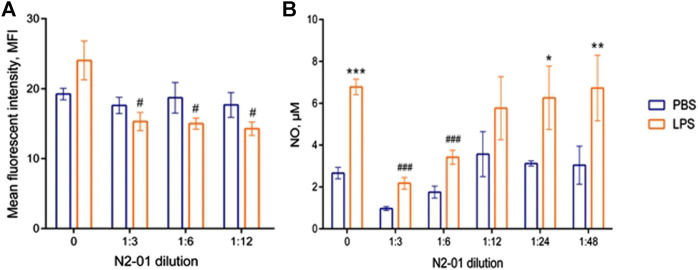
The effect of N2-01 on iNOS **(A)** expression level and NO **(B)** production by mouse peritoneal lavage cells. Statistical assessment of differences was performed using ANOVA, and pairwise comparison of the mean values was performed using the Tukey HSD test. Data are expressed as mean ± SEM of at least three individual experiments (*n* = 3). The differences are significant: a) ANOVA *p* < 0.05; # - *p* < 0.05 vs LPS; b) ANOVA *p* < 0.001; * - *p* < 0.05, *** - *p* < 0.001 vs control; ## - *p* < 0.01 vs LPS.

On the contrary, N2-01 significantly suppressed spontaneous and LPS-induced NO production by murine peritoneal lavage cells in the dilution range from 1/3 to 1/12 (Figure 5B). Besides, the drug reduced spontaneous and induced by LPS iNOS expression (Figure 5A). The results suggested that N2-01 can enhance eNOS and inhibit iNOS activity.

**FIGURE 6:**
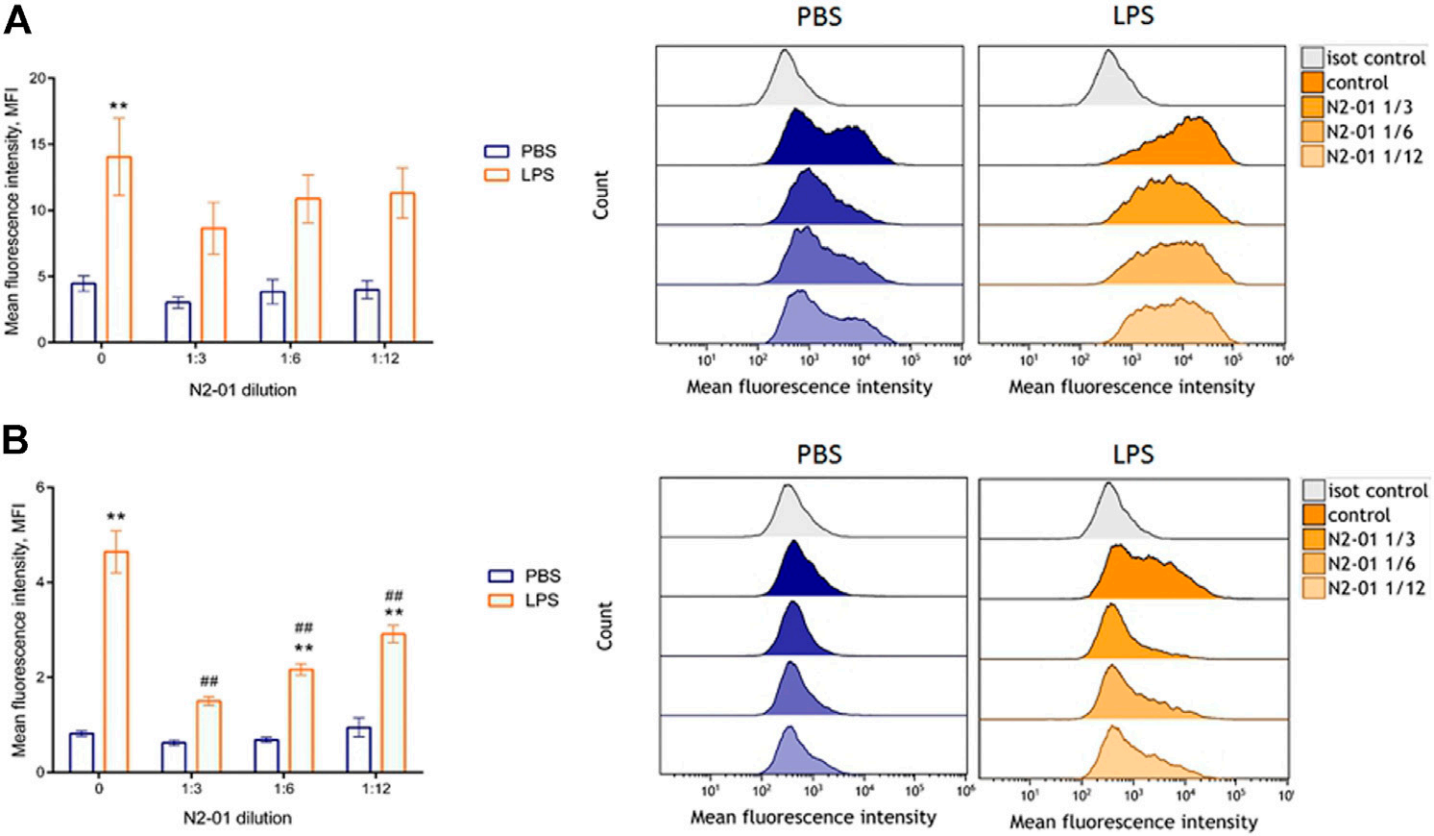
The effect of N2-01 on the ICAM-1 **(A)** and VCAM-1 **(B)** adhesion molecules expression on the human umbilical endothelial cells. Statistical assessment of differences was performed using ANOVA (*p* < 0.001), and pairwise comparison of the mean values was performed using the Tukey HSD test. Data are expressed as mean ± SEM of at least four individual experiments (*n* = 4). The differences are significant: ** - *p* < 0.01 vs control; ## - *p* < 0.01 vs LPS.

**FIGURE 7:**
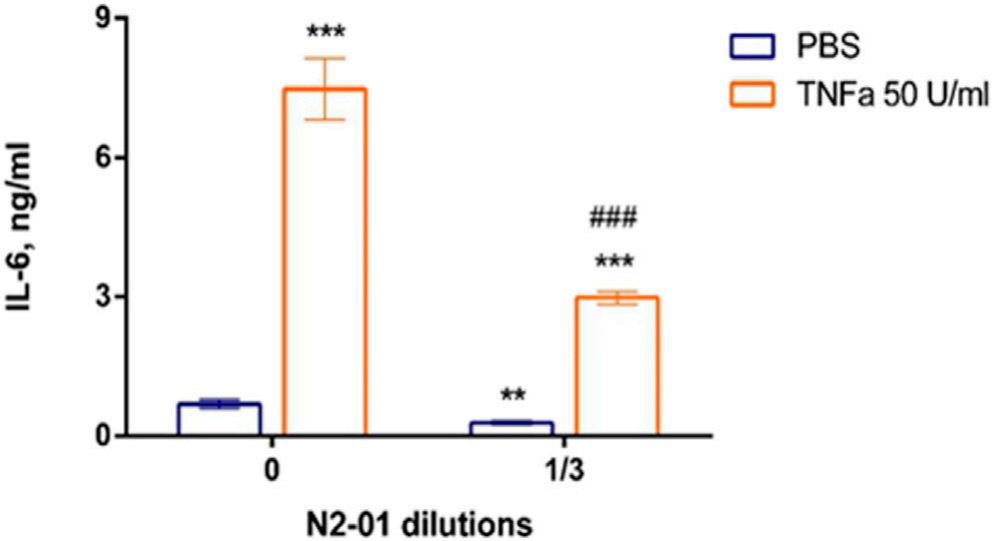
The effect of N2-01 on the IL-6 productions by human umbilical endothelial cells. Statistical assessment of differences was performed using ANOVA (*p* < 0.001), and pairwise comparison of the mean values was performed using the Tukey HSD test. Data are expressed as mean ± SEM of nine individual experiments (*n* = 9). The differences are significant: ** - *p* < 0.01 vs control, *** - *p* < 0.001 vs control; ### - *p* < 0.001 vs TNFα.

**FIGURE 8:**
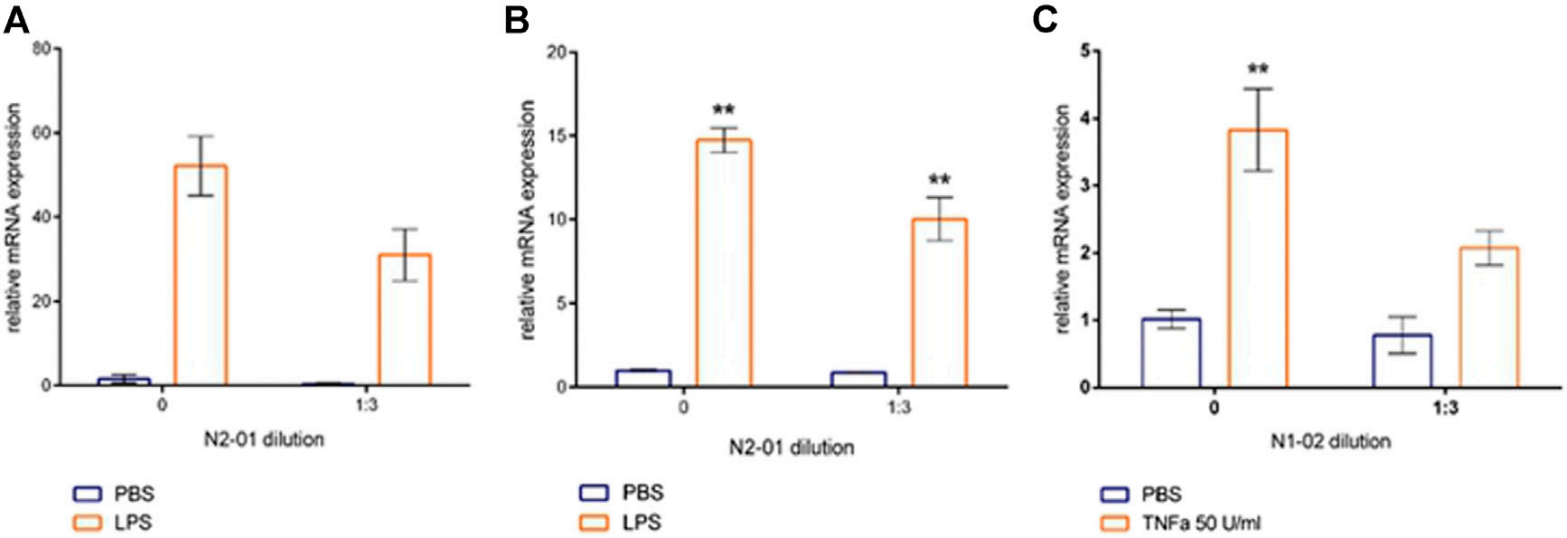
The effect of N2-01 on the VCAM-1 **(A)**, ICAM-1 **(B)** adhesion molecules and IL-6 transcripts in human umbilical endothelial cells (C). Statistical assessment of differences was performed using ANOVA (*p* < 0.001), and pairwise comparison of the mean values was performed using the Tukey HSD test. Data are expressed as mean ± SEM of three individual experiments (*n* = 3). The differences are significant: ** - *p* < 0.01 vs control.

The development of inflammatory reaction in LPS induced liver injury is accompanied by increased inflammatory cytokine productions and vascular endothelial adhesiveness (van Oosten et al., 1995). To test the potential anti-inflammatory properties of N2-01, the drug effect on the inducible adhesion molecules VCAM-1 and ICAM-1 expression on HUVECs was studied. In addition, the influence of N2-01 on IL-6 productions by endothelial cells was assessed. It was found that incubation of cells with the N2-01 in dilution 1/3 resulted in a significant decrease in the spontaneous level of VCAM-1 expression (Figure 6B). All dilutions of N2-01 showed significant reductions in the level of VCAM-1 expression induced by LPS. The drug also decreased the spontaneous and LPS induced level of ICAM-1 expression, but in this case, its effect was not statistically significant (Figure 6A). Inflammatory cytokines IL-6 and TNF-α are involved in LPS/D-GalN-induced hepatic damage (Li et al., 2018). As an essential source and target of cytokines, endothelium plays a crucial role in inflammation and amplifies tissue damage. This investigation showed that N2-01 significantly decreased IL-6 productions by resting and TNFα-activated endothelial cells (Figure 7). Further, the assessment of ICAM-1, VCAM-1 and IL-6 gene expression levels by quantitative PCR was performed. The qPCR results showed downregulation of ICAM-1, VCAM-1 and IL-6 mRNA expression under the influence of N2-01, but the effect was not statistically significant (Figure 8A–C).

### Hydrolysate Restore Endothelial Barrier Integrity

**FIGURE 9:**
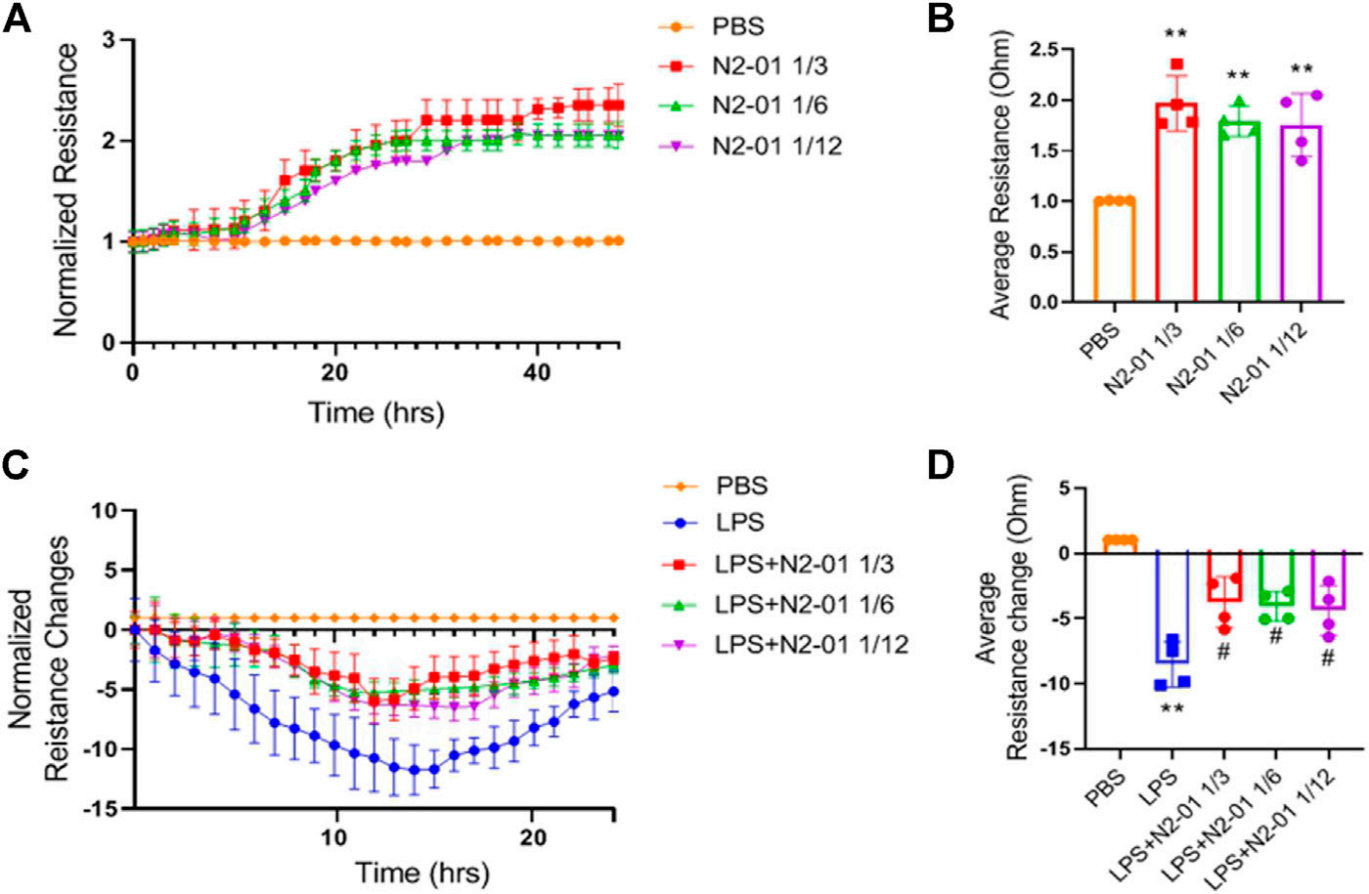
Effect of N2-01 on barrier function. Endothelial cells were pre-treated with N2-01 for 48 h and then stimulated with LPS (1 mg/ml) for 24 h. The capacity of N2-01 to restore barrier function in response to LPS has been monitored and recorded for 24 h. **(A)** Line graph showing the measurement of resistance of endothelial monolayer after N2-01 treatment at indicated doses. **(B)** Bar graphs show the data of average resistance measurements continuously recorded for and at 48 h. **(C)** Line graph showing the measurement of resistance of endothelial monolayer after LPS treatment for 24 h. **(D)** Bar graphs show the data of average resistance measurements continuously recorded for and at 24 h. Data are presented as mean ± SD of four individual experiments (n = 4). For **(B)** and **(D)**: **p < 0.01 vs PBS; #*p* < 0.05 vs LPS.

Pro-inflammatory mediators via autocrine and paracrine mechanism contribute to endothelial dysfunction, endothelial barrier integrity disruption, and extravascular fluid accumulation. Clinical observations showed that patients with sepsis usually develop progressive subcutaneous and body cavity oedema, which indicates a systemic increase in vascular permeability. Those can impair organ function by increasing the distance required for oxygen diffusion and disrupting microvascular perfusion due to increased interstitial pressure (Lee and Slutsky, 2010). Exposure to LPS induces morphological changes in endothelial cells, such as cell contraction, disruption of endothelial junctions, and loss of focal contacts with the underlying extracellular matrix, thus allowing the opening of endothelial monolayer (Bannerman and Goldblum, 1999). The response of the endothelial monolayer barrier to LPS can be assessed in real-time in a fully standardized manner by continuously recording changes in resistance changes using ECIS (Tiruppathi et al., 1992). For this assay, human brain endothelial cells were used because of the high level of tight junctions in comparison to endothelial cells from peripheric organs (Helms et al., 2016). To functionally test the effect of N2-01 on monolayer permeability after LPS stimulation, human primary brain microvascular endothelial cells grown to form a tight monolayer in 8W10E+ array slides were pre-treated with N2-01 at different dilutions, and changes in resistance of the endothelial monolayers were continuously recorded for 48 h (Figure 9A). As shown in Figure 9B, the N2-01 treatment increased the endothelial monolayer resistance. The formed endothelial monolayer was then treated with LPS (1 mg/ml), and changes in resistance were recorded for 24 h (Figure 9A). N2-01 activity on resistance became significantly evident 4 h after the beginning of treatment with LPS and persisted for more than 12 h. As indicated by the resistance values, the N2-01 treatment reduced the LPS-mediated permeability in the endothelial monolayers (Figure 9D).

**FIGURE 10:**
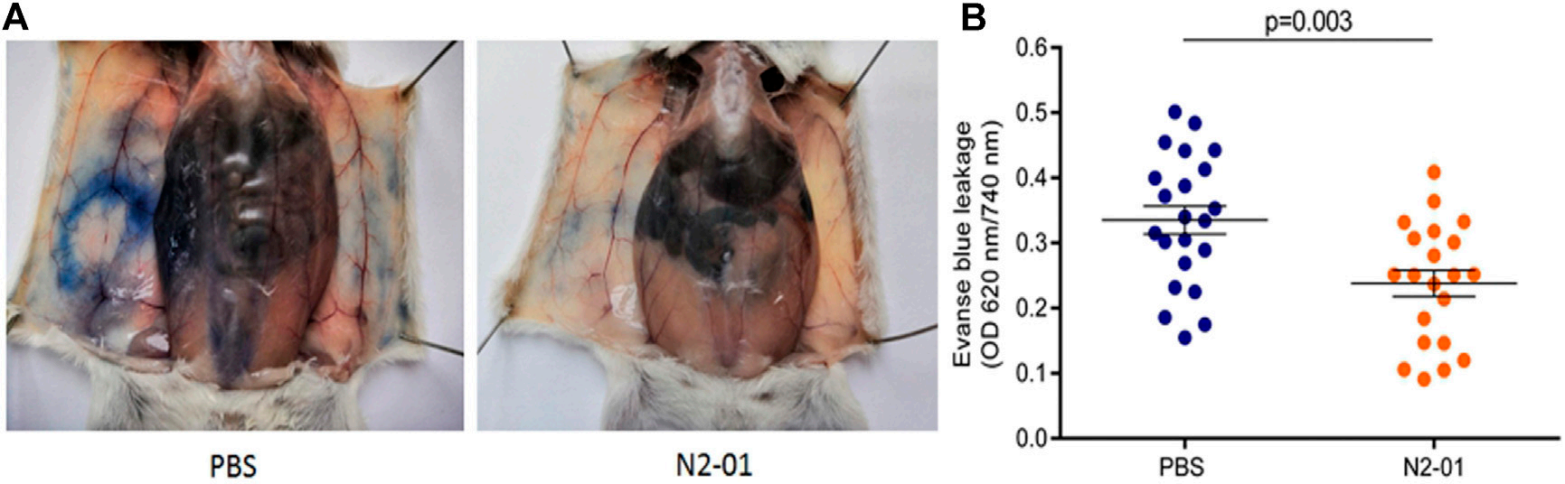
The effect of N2-01 on vascular permeability A, B. Note. Statistical assessment of differences was performed using Student`s *t*-test. The data are presented as mean ± SEM (*n* = 22).

Further, we evaluated the effect of N2-01 on extravascular protein leakage in Miles Assay *in vivo* (Ashina et al., 2015). The control group mice showed increased extravasation of Evans blue dye than the mice that were injected with N2-01 (Figure 10A). The control group mice showed increased extravasation of Evans blue dye in the tissue than the mice injected with N2-01 (Figure 10A). Quantitative analysis of Evans blue dye extracted from the mice’s skin showed that N2-01 significantly (compared to the group receiving PBS) reduced vascular permeability induced by substance 48/80 (Figure 10B).

## Discussion

In this study, evidence was obtained that N2-01, a preparation of *M. edulis* hydrolysate, significantly increases the survival rate of mice in the model of LPS/GalN acute liver injury (Figure 3). It was found that the effectiveness of the drug depends on the route of administration. While the mortality rate of mice with dosed administration of the drug was 25% (Figure 3A–C), constant administration of small doses (1.0 μl/h or 0.05 ml/kg per hour) of the drug using Mini-Osmotic Pump (Figure 3D) improved mice survival up to 100%. Based on these data, it can be assumed that due to the high rate of N2-01 catabolism, the drug’s constant administration is required to achieve the maximum positive effect.

Increased NO production is an essential pathogenetic factor that contributes to aggravating the development of acute liver injury (Guler et al., 2004). Depending on the concentration, NO can have opposite biological effects, and the metabolite production is regulated by activation of different NOS isoforms (Wink and Mitchell, 1998). LPS induces iNOS expression in leukocytes and leads to the production NO in high concentrations (>1 µm), which causes cell damage and contributes to the amplification of an inflammatory response (Sass et al., 2001). D-GalN increases animals’ sensitivity to LPS and LPS-induced production of inflammatory mediators significantly (Tang et al., 2019). Another eNOS isoform of the enzyme is constitutively expressed in endothelial cells and produces low concentrations of NO (<1 µm), essential for maintaining vascular homeostasis (Cinelli et al., 2020). Our data show that the protective effect of the N2-01 in murine model LPS/D-GalN- acute liver injury may be related to its ability to restore the balance of iNOS and eNOS activity, returning NO production to physiological values (Figures 4, 5).

Experimental and clinical data indicate that LPS/D-GalN induced toxic shock is accompanied by the overproduction of reactive oxygen species and peroxynitrite, developing severe oxidative stress with increased endothelium adhesiveness and damage (Korish and Arafa, 2011). In this study, it was also found that N2-01 decreased VCAM-1 expression (Figure 6) and IL-6 production (Figure 7) in endothelial cells, and decreased endothelium permeability (Figures 9, 10). These results confirm earlier obtained data that the >5kDa peptide fraction of *M. edulis* hydrolysate suppressed LPS-induced production of NO, prostaglandin E2 (PGE2) and pro-inflammatory cytokines TNF-α, IL-6, and IL-1β in RAW 264 macrophage cells (Park et al., 2014; Kim et al., 2016). The action of >5kDa *M. edulis* hydrolysate peptide fraction was associated with inhibition of NF-κB, MAPK signalling pathways, expression of iNOS and cyclooxygenase-2 (Park et al., 2014; Kim et al., 2016).

We hypothesis that N2-01 drug’s action is based on its ability to control the renin-angiotensin system (RAS). The primary function of RAS is traditionally considered to be the regulation of blood pressure. Recently, however, there has been evidence that RAS is also involved in the regulation of the inflammatory process. Antihypertensive classes of drugs, angiotensin-converting enzyme (ACE) and bradykinin inhibitors, are currently widely used to reduce inflammation in several diseases, such as atherosclerosis, arthritis, steatohepatitis, colitis, pancreatitis and nephritis (Ranjbar et al., 2019). It was found that a shift in the balance towards ACE2 within RAS leads to a decrease in iNOS activity, expression of adhesion molecules, production of pro-inflammatory cytokines, and restoration of the eNOS function (Ranjbar et al., 2019). Studies on rats with spontaneous hypertension showed that the hydrolysate obtained from blue mussel meat has ACE inhibitory activity (Je et al., 2005; Neves et al., 2016).

The ability of N2-01 to protect against LPS/D-GalN-induced acute liver injury could be attributed to the inhibition of inflammatory cytokines and normalization of NO production leading to the inhibition of endothelial cells adhesion molecule expression and vascular permeability. Whether the effect of N2-01 is realized by correcting the imbalance of RAS homeostasis remains to be confirmed. The anti-inflammatory effects of N2-01 established in our study make it a promising drug for use in different conditions associated with endothelial dysfunction and inflammation, such as systemic inflammatory response syndrome, oncological, neurodegenerative processes and pain syndromes.

## Data Availability

The datasets presented in this study can be found in online repositories. The names of the repository/repositories and accession number(s) can be found in the article/Supplementary Material.

## Abbreviations

ACE, angiotensin-converting enzyme; D-GalN, D-Galactosamine; FCS, fetal calf serum; HUVEC, human umbilical vein endothelial cells; ICAM-1, intercellular adhesion molecule-1; IL, interleukin; LPS, lipopolysaccharide; MIF, macrophages migration inhibiting factor; NO, nitric oxide; RAS, renin-angiotensin system; PGE2, prostaglandin E2; VCAM-1, vascular cell adhesion molecule-1.
