# Supplementary material for: Protective Role of Mytilus edulis Hydrolysate in Lipopolysaccharide-Galactosamine Acute Liver Injury
Source: Front Pharmacol. 2021 May 18;12:667572. doi: 10.3389/fphar.2021.667572 (PMC8167060; doi:10.3389/fphar.2021.667572)
Supplement: Supplementary file 3 [file Table3.DOCX]

*# R-code for generating heatmap from Compound Discoverer output.*

cdcs <- read.delim("Compounds.tsv")

i<-grep("Repl.*raw",colnames(cdcs))

colnames(cdcs)[i]

x <- cdcs[complete.cases(cdcs[i]),i]

x$names<-cdcs$Name[complete.cases(cdcs[i])]

**library**(reshape2)

m <- melt(x[x$names!="",])

xm <- dcast(m, names ~ variable, sum)

xc <- xm[complete.cases(xm),]

rownames(xc) <- xc$names

xc$names<-**NULL**

colnames(xc) <- gsub(".*_","",colnames(xc))

**library**(RColorBrewer)

heatmap(scale(log10(xc[,c(13,15,16,18,19,21,22,24,25,27)])),labRow = "",

col= colorRampPalette(brewer.pal(8, "Oranges"))(25))

legend(x="bottomright",legend=c("min","ave","max"),

fill= colorRampPalette(brewer.pal(8, "Oranges"))(3))
